# Monitoring disease activity in multiple sclerosis using serum neurofilament light protein

**DOI:** 10.1212/WNL.0000000000004683

**Published:** 2017-11-28

**Authors:** Lenka Novakova, Henrik Zetterberg, Peter Sundström, Markus Axelsson, Mohsen Khademi, Martin Gunnarsson, Clas Malmeström, Anders Svenningsson, Tomas Olsson, Fredrik Piehl, Kaj Blennow, Jan Lycke

**Affiliations:** From the Department of Clinical Neuroscience (L.N., M.A., C.M., J.L.) and Department of Psychiatry and Neurochemistry (H.Z., K.B.), Institute of Neuroscience and Physiology at Sahlgrenska Academy, University of Gothenburg; Clinical Neurochemistry Laboratory (H.Z., K.B.), Sahlgrenska University Hospital, Mölndal, Sweden; Department of Molecular Neuroscience (H.Z.), UCL Institute of Neurology, London, UK; Department of Pharmacology and Clinical Neuroscience (P.S.), Umeå University; University Department of Clinical Neuroscience (M.K., T.O., F.P.), Neuroimmunology Unit, and Department of Clinical Sciences (A.S.), Danderyd Hospital, Karolinska Institutet, Stockholm; and Department of Neurology (M.G.), Faculty of Medicine and Health, Örebro University, Sweden.

## Abstract

**Objective:**

To examine the effects of disease activity, disability, and disease-modifying therapies (DMTs) on serum neurofilament light (NFL) and the correlation between NFL concentrations in serum and CSF in multiple sclerosis (MS).

**Methods:**

NFL concentrations were measured in paired serum and CSF samples (n = 521) from 373 participants: 286 had MS, 45 had other neurologic conditions, and 42 were healthy controls (HCs). In 138 patients with MS, the serum and CSF samples were obtained before and after DMT treatment with a median interval of 12 months. The CSF NFL concentration was measured with the UmanDiagnostics NF-light enzyme-linked immunosorbent assay. The serum NFL concentration was measured with an in-house ultrasensitive single-molecule array assay.

**Results:**

In MS, the correlation between serum and CSF NFL was *r* = 0.62 (*p* < 0.001). Serum concentrations were significantly higher in patients with relapsing-remitting MS (16.9 ng/L) and in patients with progressive MS (23 ng/L) than in HCs (10.5 ng/L, *p* < 0.001 and *p* < 0.001, respectively). Treatment with DMT reduced median serum NFL levels from 18.6 (interquartile range [IQR] 12.6–32.7) ng/L to 15.7 (IQR 9.6–22.7) ng/L (*p* < 0.001). Patients with relapse or with radiologic activity had significantly higher serum NFL levels than those in remission (*p* < 0.001) or those without new lesions on MRI (*p* < 0.001).

**Conclusions:**

Serum and CSF NFL levels were highly correlated, indicating that blood sampling can replace CSF taps for this particular marker. Disease activity and DMT had similar effects on serum and CSF NFL concentrations. Repeated NFL determinations in peripheral blood for detecting axonal damage may represent new possibilities in MS monitoring.

Neurofilament light (NFL) protein is one of the most studied biomarkers of disease activity and treatment response in patients with multiple sclerosis (MS). Neurofilaments are structural components of myelinated axons that are composed of subunits known as light, medium, heavy, α-internexin, and peripherin. Neurofilaments are released into the CSF after axonal injury^1^ during various neurologic disorders, including MS.^2^

In MS, the concentration of CSF NFL is increased during relapse and in conjunction with contrast-enhancing lesions on MRI.^3–6^ The concentration is decreased by effective treatment with disease-modifying therapies (DMTs).^3,4,7^ The CSF NFL concentration at disease onset may predict disease severity.^8,9^ The sensitivity of the immunoassay has been improved, making it possible to determine NFL levels as low as those found in the CSF of healthy controls (HCs).^1^

Recent technical developments have given rise to ultrasensitive antibody-based analytic techniques such as the single-molecule array (Simoa) technology, which enables quantification of protein biomarkers in blood samples at very low concentrations.^10^ We recently developed a Simoa method for NFL in blood samples (serum or plasma) that has markedly improved analytic sensitivity compared to standard enzyme-linked immunosorbent assay (ELISA) or electrochemiluminescence immunoassays, allowing accurate measurement of NFL in blood down to concentrations occurring in healthy persons.^11^ Using the Simoa assay, blood NFL has shown promise as a biomarker for HIV encephalopathy,^12^ severe traumatic brain injury,^13^ sports-related mild traumatic brain injury,^14^ postconcussion syndrome,^15^ and MS.^16^

Currently, we are still lacking a reliable blood biomarker for evaluating CNS injury in MS. In this study, NFL concentrations were measured in 521 paired serum and CSF samples collected at 4 university hospitals in Sweden. The aims were to investigate the correlation between serum and CSF NFL concentrations and to investigate the effects of disease activity, disability, and DMTs on serum NFL concentrations in MS.

## METHODS

### Patients and HCs.

Patients with MS (n = 286) fulfilling the revised McDonald criteria,^17^ patients with other neurologic disorders or symptoms (ONDs, n = 45), and HCs (n = 42)^18^ were consecutively enrolled in the study at the neurology departments of 4 Swedish hospitals: Umeå University Hospital, Umeå; Sahlgrenska University Hospital, Gothenburg; Karolinska University Hospital, Stockholm; and Örebro University Hospital, Örebro (table).

**Table:**
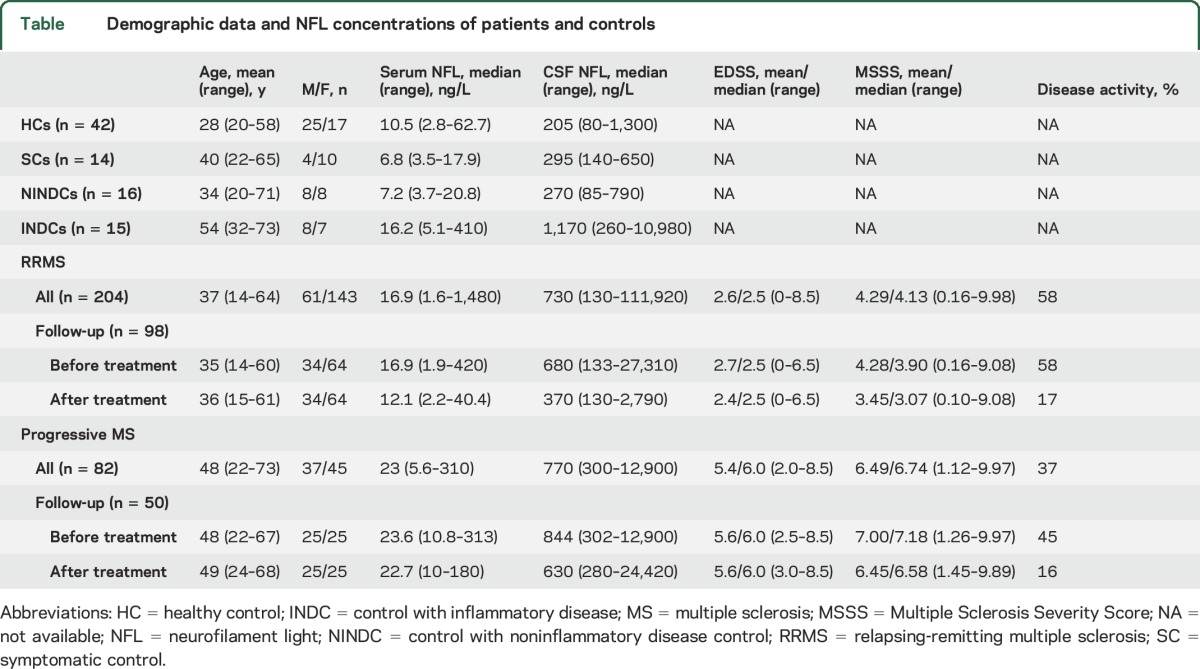
Demographic data and NFL concentrations of patients and controls

The 286 patients with MS consisted of 204 with relapsing-remitting MS (RRMS) and 82 with progressive MS; the latter group included 19 with primary progressive MS and 63 with secondary progressive MS. A subgroup of these patients (n = 148), including 98 with RRMS and 50 with progressive MS, were followed up prospectively and examined before and again after a median of 12 months (range 0–46 months). A small proportion remained untreated (n = 10), and the remaining (n = 138) were treated with the following DMTs: glatiramer acetate (n = 2), glatiramer acetate plus mitoxantrone (n = 1), interferon-β (n = 3), oral weekly methotrexate (n = 7), mitoxantrone (n = 15), fingolimod (n = 21), rituximab (n = 23), natalizumab (n = 63), alemtuzumab (n = 2), and cyclophosphamide (n = 1). In conjunction with baseline sampling, most patients escalated their DMT (n = 68) from less effective DMTs (interferon-β, glatiramer acetate, high doses of IV immunoglobulin, or oral weekly methotrexate) to more effective DMTs (alemtuzumab, cyclophosphamide, fingolimod, glatiramer acetate plus mitoxantrone, mitoxantrone, natalizumab, or rituximab).^19^ A second group of patients were not being treated at the time of sampling and either were treatment naive (n = 36) or had a prior treatment terminated >3 months previously (n = 14). A third group (n = 20) had changed to another DMT with similar efficacy because of adverse effects or unsatisfactory adherence.

Participants with ONDs served as controls and were divided according to established definitions^18^ into those with inflammatory neurologic diseases (INDCs, n = 15), including CNS vasculitis (n = 2), giant cell arteritis (n = 1), antiphospholipid antibody syndrome (n = 1), systemic lupus erythematosus (n = 1), neuroborreliosis (n = 1), sarcoidosis (n = 3), chronic lymphatic leukemia with CNS involvement (n = 1), myelitis (n = 1), neuromyelitis optica spectrum disorders (n = 1), and unspecified demyelinating disease (n = 3); those with noninflammatory neurologic diseases (n = 16), including psychosis (n = 14), epilepsy (n = 1), and Horner syndrome (n = 1); and symptomatic controls (n = 14), including those with sensory symptoms (n = 6), headache (n = 2), dizziness (n = 1), fatigue (n = 2), visual disturbance (n = 1), and unspecified neurologic symptoms (n = 2).

Healthy blood donors and university students served as HCs (n = 42). None of the HCs had any neurologic signs or history of neurologic disease.

### Clinical assessments and MRI.

Patients were assessed once (n = 138) or were followed up prospectively and assessed twice (n = 148) by clinical neurologic examination performed by MS-specialized neurologists. Disability was scored by the Expanded Disability Status Scale (EDSS),^20^ and disease severity was scored by the Multiple Sclerosis Severity Score.^21^ A relapse was defined as an episode of neurologic disturbance lasting for at least 24 hours that could not be better explained by another cause.^22^

A standard MRI protocol for MS with IV gadolinium (Gd) as contrast was used. Because Gd enhancement on MRI appears in the majority of cases during a period of up to 6 weeks (mean 3.07 weeks),^17^ we chose to include only MRIs performed 6 weeks before or after lumbar puncture and peripheral blood test (n = 324) to investigate the influence of disease activity on MRI. The disease activity was defined as a relapse or Gd-enhancing lesion. Because of the absence of data for T2 lesions on MRI, we used a modified no evidence of disease activity (NEDA)^23^: absence of contrast-enhancing lesions on MRI, absence of confirmed disability progression defined as the absence of increased posttreatment disability by 1.0 if the EDSS score was 0 to 5.5 at baseline or by 0.5 if the EDSS score was ≥6.0 at baseline, and absence of relapses.^24^

### Blood tests and CSF sampling.

Samples of peripheral blood and CSF were obtained at the clinical assessments. The CSF samples were handled according to the consensus protocol of the BioMS-EU network for CSF biomarker research in MS.^25^ In patients with MS, the sampling period was dichotomized between relapse and remission. The relapse period was the time between relapse onset and 3 months later because increased concentrations of NFL are expected within this period of time.^5,16^

### NFL analysis.

All measurements were performed by board-certified laboratory technicians in the Clinical Neurochemistry Laboratory at the Sahlgrenska University Hospital, i.e., by laboratory technicians who are licensed to perform clinical laboratory measurements by the National Board of Health and Welfare, a government agency in Sweden under the Ministry of Health and Social Affairs.

The concentration of NFL in CSF was measured with a sensitive sandwich ELISA method (NF-light ELISA kit; UmanDiagnostics AB, Umeå, Sweden) according to the ELISA kit instructions. The lower limit of quantification (LLoQ) of the assay was 31 ng/L. The intra-assay and interassay coefficients of variation were <10%.

The concentration of NFL in serum was determined with the NF-light assay, which was adapted for the Simoa platform with a Homebrew Kit (Quanterix Corp, Boston, MA). The LLoQ, which was determined by the blank mean signal at 610 SD, was 1.95 ng/L. All samples were measured in duplicate and were well above the LLoQ. The intra-assay and interassay coefficients of variation were <10%. The method is described in detail elsewhere.^26^

### Statistical analysis.

Statistical calculations were performed with IBM SPSS Statistics 21 software (IBM Corp, Armonk, NY). Because of the nonnormal distribution of serum and CSF NFL levels, the analyses were performed with nonparametric tests, the Kruskal-Wallis test for comparison of multiple groups, and the Mann-Whitney test for comparison of 2 groups. The results are presented as median NFL levels and interquartile range. Correlations between serum and CSF NFL levels were analyzed with the Spearman rank correlation coefficient. The receiver operating characteristic (ROC) curve estimation was performed with the assumption of nonparametric distribution. The sensitivity and specificity were calculated by the Youden index, expressed as sensitivity + specificity −1, to calculate optimal cutoffs that maximize both sensitivity and specificity.

### Standard protocol approvals, registrations, and patient consents.

All patients and controls participated voluntarily in the study and provided written informed consent. The regional ethics review boards in Uppsala and Stockholm, Sweden, approved the study.

## RESULTS

### Comparison of serum and CSF NFL levels in patients with MS and controls.

A total of 521 paired CSF and serum samples were analyzed. Serum and CSF NFL concentrations were significantly higher in patients with MS than in HCs (*p* < 0.001 and *p* < 0.001, respectively) and in controls with noninflammatory neurologic disease and symptomatic controls (*p* < 0.001 and *p* < 0.001, respectively). No difference was found between serum NFL concentrations in INDCs vs patients with MS, whereas CSF NFL concentrations were higher in INDCs than in patients with MS (*p* = 0.019, table). NFL concentrations in serum and CSF were higher in patients with RRMS with disease activity than in those without disease activity (*p* < 0.001 and *p* < 0.001, respectively). NFL concentrations in serum and CSF were also higher in patients with progressive MS with disease activity than in those without activity (*p* = 0.009 and *p* < 0.001, respectively; table).

### Effect of treatment on NFL levels in patients with MS.

**Figure 1:**
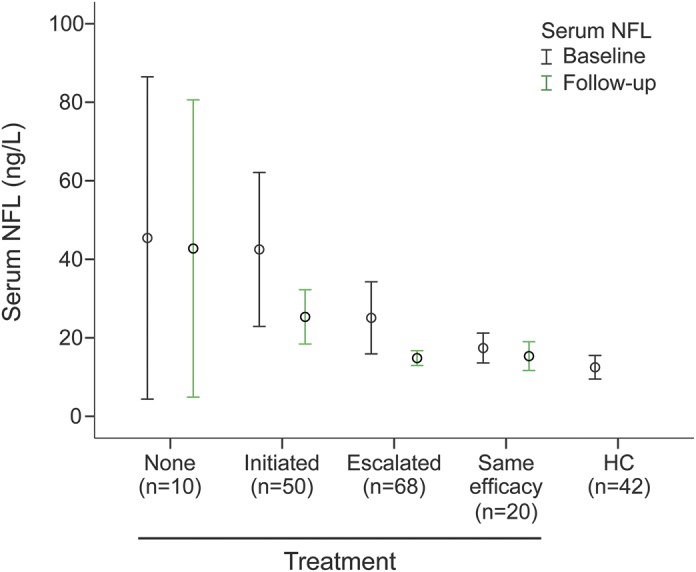
Serum NFL concentrations in patients with MS at baseline and follow-up and in HCs Serum NFL concentrations at baseline and follow-up in patients with MS who remained untreated, in patients with MS who initiated treatment with DMTs, in patients with MS who escalated DMT to more effective therapy, in patients with MS who changed treatment between DMTs of similar efficacy, and in HCs. The figure shows median and 95% confidence interval of serum NFL concentrations. DMT = disease-modifying therapy; HC = healthy control; MS = multiple sclerosis; NFL = neurofilament light.

In untreated patients who initiated DMT, median NFL concentrations in serum decreased from 22.7 (17.5–39.1) to 20.2 (13.7–28.9) ng/L (*p* = 0.002), and CSF NFL concentrations decreased from 907 (564–1,608) to 460 (350–675) ng/L (*p* < 0.001). In patients who escalated their DMT to more effective therapy, serum NFL concentrations decreased from 17.7 (11.8–25.6) to 12.4 (8.3–19.7) ng/L (*p* < 0.001), and CSF NFL concentrations decreased from 650 (406–1,220) to 376 (242–623) ng/L (*p* < 0.001). The patients who remained untreated at follow-up had unchanged serum and CSF NFL concentrations between the sampling time points: 26.1 (12.1–52) vs 26 (11.6–53.8) ng/L (*p* = 0.515) for serum and 1,003 (631–1,529) vs 600 (451–1,177) ng/L (*p* = 0.285) for CSF. Patients who changed treatment between DMTs with similar efficacy had stable serum and CSF NFL concentrations between the sampling time points: 15.7 (11–21.1) vs 15.4 (9.1–18.3) ng/L (*p* = 0.247) for serum and 565 (375–863) vs 464 (338–660) ng/L (*p* = 0.086) for CSF (figure 1).

### Relationship of NFL levels to disability, disease severity, and clinical and radiologic disease activity.

Patients with a relapse (n = 86) within 3 months before sampling had higher NFL concentrations in serum of 19.1 (12.4–38.3) ng/L and in CSF of 925 (478–2,155) ng/L than patients in remission (n = 346), who had NFL concentrations in serum of 17.2 (11.4–25.4) ng/L and in CSF of 570 (370–927) ng/L (*p* = 0.043 and *p* < 0.001, respectively). CSF NFL and serum NFL levels against the time from relapse onset are shown in figures e-1 and e-2 at Neurology.org. Serum and CSF NFL concentrations correlated weakly with EDSS (ρ = 0.380, 95% confidence interval [CI] 0.297–0.457, *p* < 0.001; and ρ = 0.243, 95% CI 0.153–0.329, *p* < 0.001, respectively) and with Multiple Sclerosis Severity Score (ρ = 0.392, 95% CI 0.310–0.468, *p* < 0.001; and ρ = 0.340, 95% CI 0.255–0.420, *p* < 0.001, respectively).

**Figure 2:**
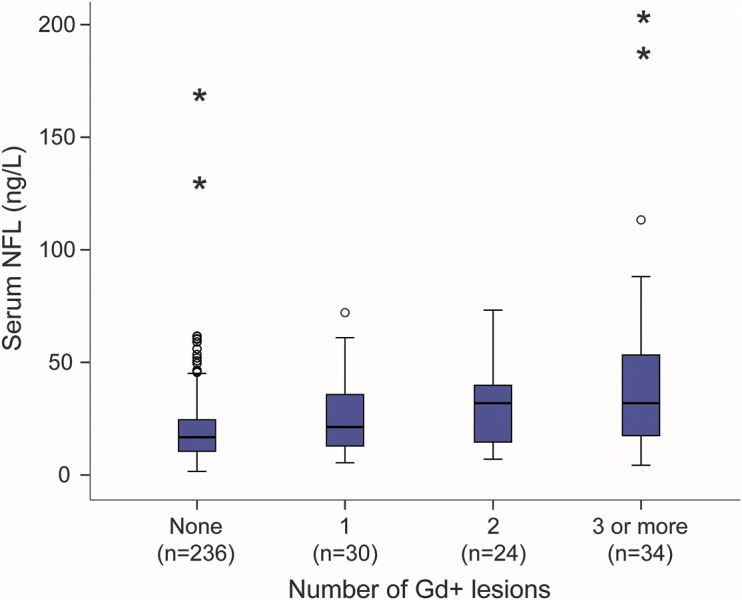
Serum NFL concentrations in patients with MS with different numbers of Gd-enhancing lesions Serum NFL concentration in patients with no Gd-enhancing lesions (n = 236) was 16.8 (IQR 10.5–24.6) ng/L, with 1 Gd-enhancing lesion (n = 30) was 21.3 (IQR 12.8–36.5) ng/L, with 2 Gd-enhancing lesions (n = 24) was 31.9 (IQR 14.5–39.9) ng/L in serum, and with ≥3 Gd-enhancing lesions (n = 34) was 31.9 (IQR 17.4–55.6) ng/L. Box indicates IQR; bar indicates median, and whiskers indicate 95% confidence interval. Extreme values are marked with open dots (±1.5 × IQR) or with asterisks (±3 × IQR). IQR = interquartile range; MS = multiple sclerosis; NFL = neurofilament light.

Patients with Gd-enhancing lesions (n = 88) had higher serum and CSF NFL concentrations (22.8 [14.7–41.3] and 1,187 [708–2,166] ng/L) than patients without Gd-enhancing lesions (n = 236, 16.8 [10.5–24.6] and 499 [330–795] ng/L, *p* < 0.001 and *p* < 0.001, respectively). Serum and CSF NFL concentrations increased with the number of Gd-enhancing lesions (figure 2). Among the treated patients fulfilling the modified NEDA (n = 77), 81.3% had normal levels of CSF NFL and 67.5% had normal levels of serum NFL.

### Correlation between serum and CSF NFL concentrations, possible confounding factors, and sensitivity and specificity of serum NFL concentrations for disease activity.

**Figure 3:**
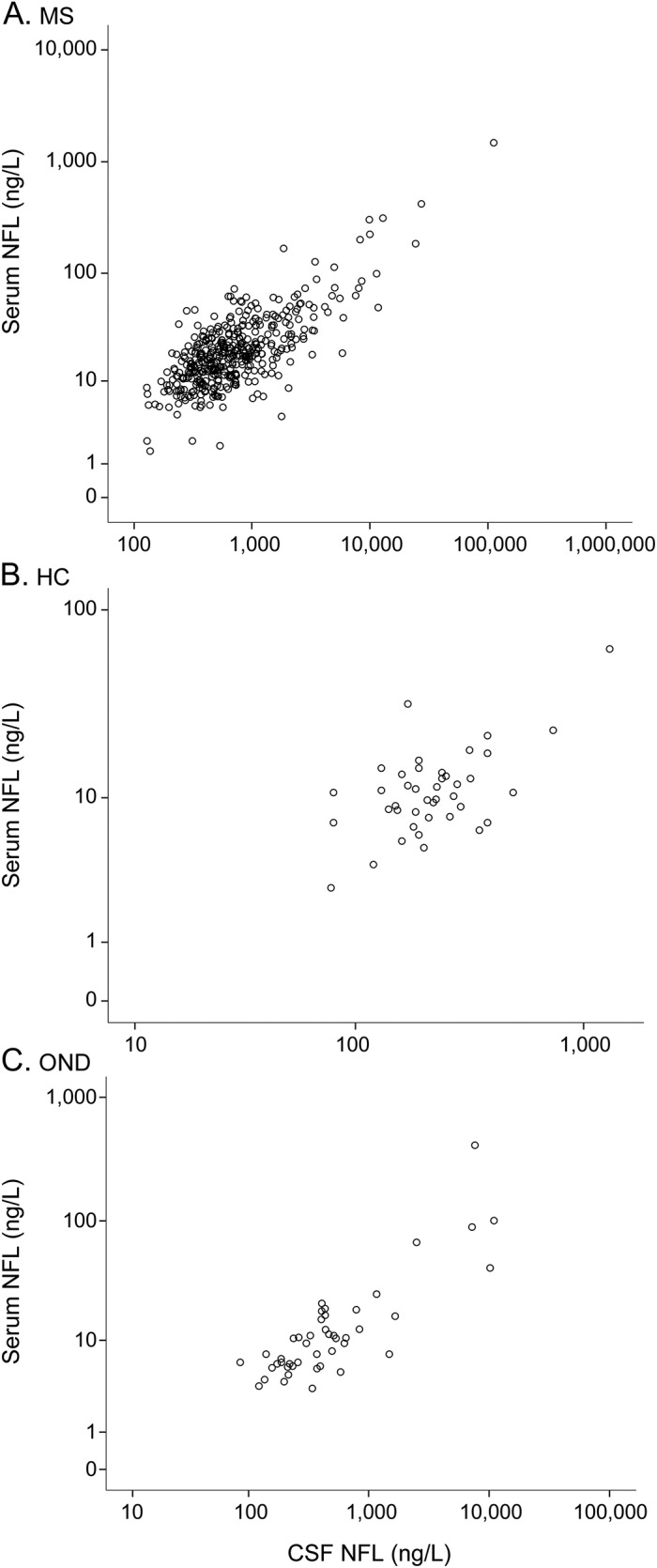
Correlation between serum and CSF NFL in patients with MS, HCs, and patients with ONDs Correlation between NFL concentrations in serum and CSF (A) in patients with MS was ρ = 0.620 (95% CI 0.558–0.675, *p* < 0.001), (B) in HCs was ρ = 0.385 (95% CI 0.092–0.616, *p* < 0.001), and (C) in patients with OND was ρ = 0.740 (95% CI 0.571–0.849, *p* < 0.001). CI = confidence interval; HC = healthy control; MS = multiple sclerosis; NFL = neurofilament light; OND = other neurological disorder or symptom.

Correlations between serum and CSF NFL were ρ = 0.620 (95% CI 0.558–0.675, *p* < 0.001) for patients with MS, ρ = 0.385 (95% CI 0.092–0.616, *p* < 0.001) for HCs, and ρ = 0.740 (95% CI 0.571–0.849, *p* < 0.001) for patients with OND (figure 3, A–C). Disease duration, age, and sex did not significantly influence CSF or serum NFL concentrations. In 158 patients with MS, CSF/serum albumin ratio, which is a biomarker for blood-brain barrier integrity, did not correlate with NFL concentration.

The arbitrary cutoff value for increased NFL concentration in serum was defined as 18.2 ng/L, which is 2 SDs above the mean NFL concentration in HCs. The age-dependent changes of CSF NFL were calculated by subtracting the expected level for the given age following the linear relationship reported in healthy individuals (i.e., 11.8 ng/L × age − 95 ng/L)^27^ from the measured NFL level.

**Figure 4:**
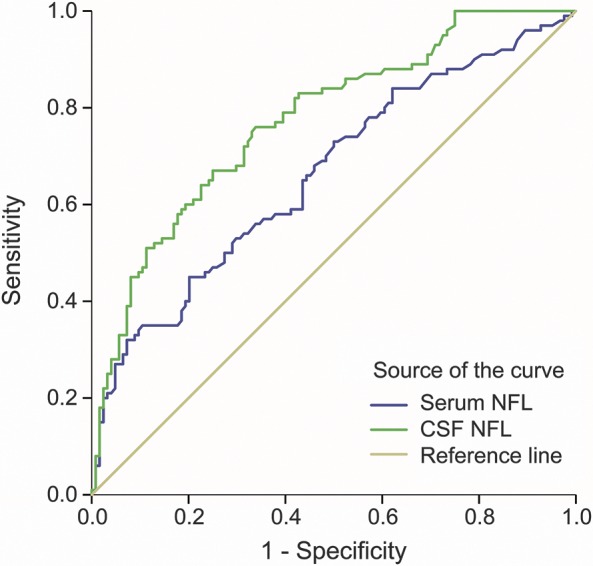
ROC curve showing specificity and sensitivity of NFL in serum and CSF for disease activity ROC curve with AUC for NFL in serum and CSF indicating specificity and sensitivity to discriminate patients with MS with disease activity from patients with MS without disease activity. AUC for serum NFL was 0.663 (95% CI 0.591–0.735, 80% specificity and 45% sensitivity) and for CSF NFL was 0.774 (95% CI 0.714–0.835, 75% specificity and 67% sensitivity). AUC = area under the curve; CI = confidence interval; MS = multiple sclerosis; NFL = neurofilament light; ROC = receiver operating characteristic.

The number of patients with RRMS with disease activity, along with the number of patients with RRMS with elevated NFL concentrations in serum and in CSF, was used to calculate sensitivity and specificity. Patients with progressive MS were excluded because increased NFL concentration in this group may be due to other degenerative processes and may not be confined to inflammatory activity. Patients with RRMS without disease activity had normal serum NFL concentrations in 93.4% of the cases and normal CSF NFL concentrations in 80% of the cases. To evaluate the value of increased NFL concentration in serum and CSF as a screening test for disease activity in RRMS, a ROC curve was constructed (figure 4). The area under the curve (AUC) for serum NFL was 0.663 (95% CI 0.591–0.735, 80% specificity and 45% sensitivity), and the AUC for CSF NFL was 0.774 (95% CI 0.714–0.835, 75% specificity and 67% sensitivity).

In patients with RRMS, 6.8% of serum samples and 32.4% of CSF samples were higher than the NFL cutoff level for those on higher-efficacy DMTs; the corresponding values in patients with RRMS on less efficacious DMTs were 12.6% and 37.8%.

## DISCUSSION

The data in this study are based on a large set of paired serum and CSF samples from a real-life cohort of patients across a wide clinical and therapeutic spectrum. They support serum NFL as a biomarker for monitoring disease activity and treatment intervention in MS. We found that serum and CSF NFL concentrations were highly correlated and reacted similarly during the different stages of MS and in response to treatment with DMTs. High serum and CSF NFL concentrations were associated with relapse and with the number of contrast-enhancing lesions on MRI. This was not confined to RRMS but was also found in patients with progressive disease course.

The effect of DMT on serum and CSF NFL concentrations was evaluated in all patients with MS regardless of whether the patients were treatment naive or on DMT at baseline. We confirmed that CSF NFL concentrations remained stable in patients who remained untreated or who switched treatment to DMT with similar efficacy, and NFL decreased in patients after initiating DMT or switching from first-line to second-line DMT.^4,7^ This response was also valid for serum NFL. Thus, the DMT efficacy was reflected by NFL concentrations, and serum NFL was as reliable as CSF NFL. Moreover, in patients with RRMS who were treated with effective DMT, we confirmed our previous finding in CSF that the NFL concentration in serum was not different from that in HCs.^4,7^

Because of its high specificity, normal concentration of serum NFL could be a useful measure for surveillance of subclinical activity in RRMS. Thus, a normal serum NFL concentration argues strongly against ongoing disease activity. In contrast, increased NFL concentrations occurred in patients who were clinically stable and who did not have contrast-enhancing lesions on cerebral MRI. New T2 lesion formation may influence the NFL concentration,^28,29^ and the absence of T2 lesion data in our material probably affected the sensitivity of serum NFL to detect disease activity. However, spinal cord lesions, diffuse tissue injury of normal-appearing white matter,^2^ and gray matter pathology^30^ may also contribute to axonal injury. Thus, serum NFL may reveal asymptomatic ongoing axonal injury that is not seen on cerebral MRI.

NFL determination can detect axonal damage that occurred up to 3 months before sampling.^5,6^ Again, the high correlation between serum and CSF NFL suggests that the temporal course of serum NFL is similar to that described for CSF NFL.^5,6,16^ However, this has to be further investigated in prospective studies. In monitoring of the effect of DMT on axonal damage, a 3-month interval between blood tests for monitoring serum NFL would reveal the occurrence of new disease activity.^4,5,16^ However, we cannot determine from our data whether this would detect a stepwise accumulation of T2 lesions, accumulation of disability, or conversion to a progressive disease course. There is a need for long-term follow-up studies to collect data on the correlation between NFL concentrations over time and such outcomes.

The NFL concentration is related to the magnitude and rate of axonal damage and does not indicate the nature of the pathologic process. Compared with clinical measures and MRI, NFL determinations add new information that other methods may not be able to reveal. Even if fulfilling the modified NEDA, a proportion of patients still had elevated levels of NFL. Thus, NFL may contribute to NEDA for accessing disease-free status, and NFL and other body fluid biomarkers as complements to current clinical and MRI measures might improve the assessment of disease activity in MS.^31^

Although there is robust evidence supporting the CSF NFL concentration as a clinically useful biomarker, the need for lumbar puncture constitutes a major barrier for more widespread use, especially when repeat lumbar punctures are needed. Here, we show that serum and CSF NFL levels were highly correlated in MS and that this relationship also was present in HCs, i.e., throughout the entire detection range of the assays. The different degree of correlation in previous studies^11,12,16^ compared to this study probably depends on the use of various clinical materials and statistical methods, i.e., parametric tests or log-transformed data. We also confirmed the high correlation between serum and CSF NFL levels in patients with OND as in previous studies on traumatic injury^15^ and HIV encephalitis.^12^

Because of the multiple treatment options, it is increasingly important to accurately identify patients with MS with insufficient treatment response. Our data suggest that measuring serum NFL may be useful in trials and in clinical practice for evaluating the effect of DMTs in MS. Thus, in combination with clinical and MRI monitoring, serum NFL can add valuable new information that will facilitate the monitoring of disease activity and treatment decisions in MS.

## Supplementary Material

Data Supplement

## GLOSSARY

AUC: area under the curve
CI: confidence interval
DMT: disease-modifying therapy
EDSS: Expanded Disability Status Scale
ELISA: enzyme-linked immunosorbent assay
HC: healthy control
INDC: control with inflammatory neurologic disease
LLoQ: lower limit of quantification
MS: multiple sclerosis
NEDA: no evidence of disease activity
NFL: neurofilament light
OND: other neurologic disorder or symptom
ROC: receiver operating characteristic
RRMS: relapsing-remitting multiple sclerosis
Simoa: single-molecule array
