# Supplementary material for: Monitoring disease activity in multiple sclerosis using serum neurofilament light protein
Source: Neurology. 2017 Nov 28;89(22):2230–7. doi: 10.1212/WNL.0000000000004683 (PMC5705244; doi:10.1212/WNL.0000000000004683)
Supplement: Data Supplement [file supp_89_22_2230__index.html]

Monitoring disease activity in multiple sclerosis using serum neurofilament light protein — Data Supplement 

# Monitoring disease activity in multiple sclerosis using serum neurofilament light protein

## Data Supplement

**Neurology® data supplements are not copyedited before publication. Published editorials and translations have been copyedited.  
 © 2017 American Academy of Neurology.  
  
 Files in this Data Supplement:**

- Figure e-1 - PDF
- Figure e-2 - PDF
