# Supplementary material for: Monitoring disease activity in multiple sclerosis using serum neurofilament light protein
Source: Neurology. 2017 Nov 28;89(22):2230–7. doi: 10.1212/WNL.0000000000004683 (PMC5705244; doi:10.1212/WNL.0000000000004683)
Supplement: Data Supplement [file supp_WNL.0000000000004683_Figure_e-1.pdf]

Figure e-1. CSF NFL

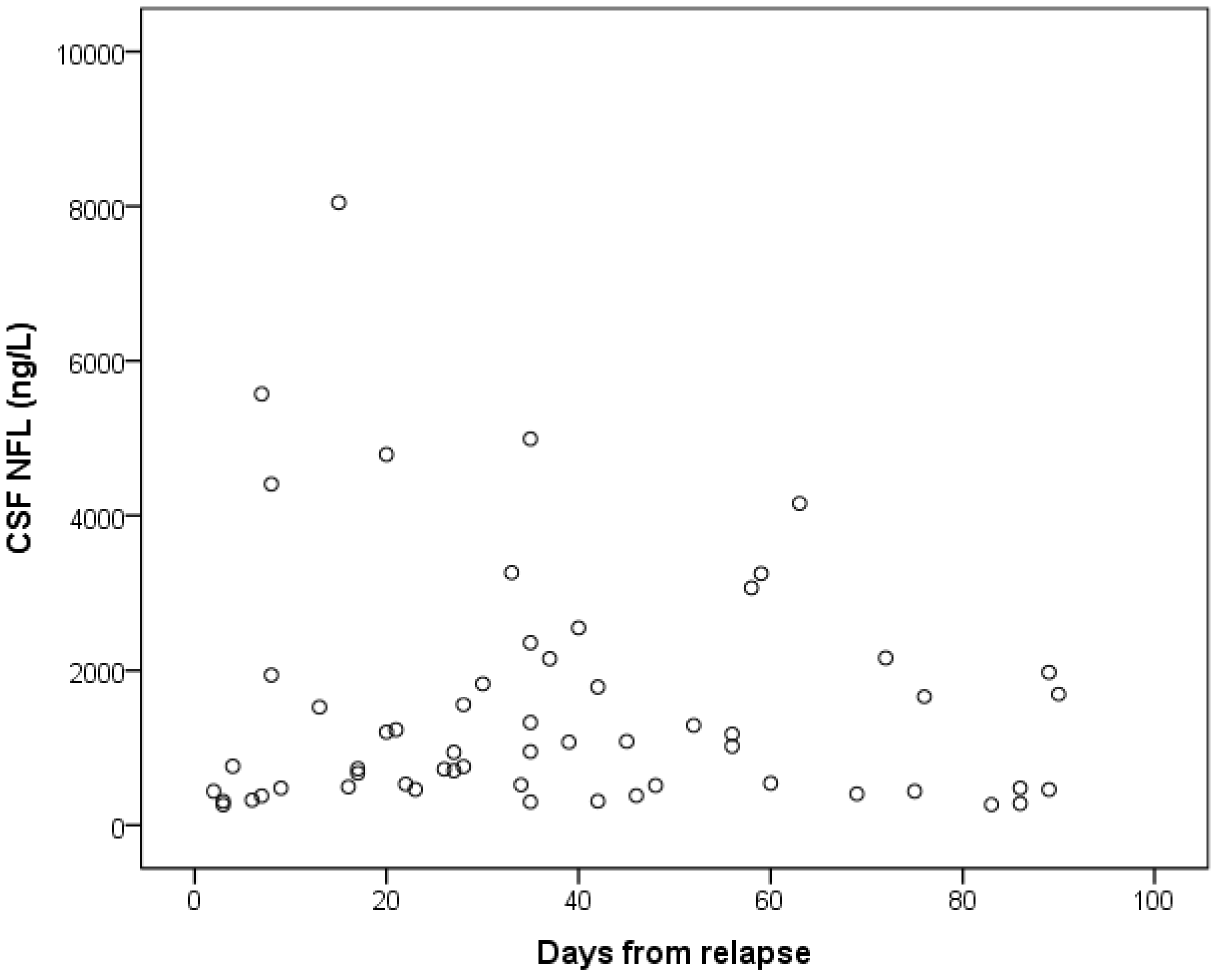

In a subgroup of the study population the timepoint of relapse onset and the NFL levels in CSF and serum are shown. These samples were obtained only once from different patients with different treatments. This explains the wide variation of NFL concentrations, also in samples obtained at similar timepoints from relapse onset. We did not follow the patients prospectively with repeated sampling from the relapse onset so we could not show the change in individual NFL levels after relapse onset. In the previous study, CSF was obtained as part of a RCT trial of acyclovir/placebo treatment in RRMS.5 In such controlled and homogenous conditions, the dynamic change of CSF NFL levels after relapse onset was shown5 and similar results have been reported for serum NFL after neurosurgical trauma.16
