# Supplementary figures and images for: Monitoring disease activity in multiple sclerosis using serum neurofilament light protein
Source: Neurology. 2017 Nov 28;89(22):2230–7. doi: 10.1212/WNL.0000000000004683 (PMC5705244; doi:10.1212/WNL.0000000000004683)

**Figure e-2. Serum NFL levels against the time from relapse onset**

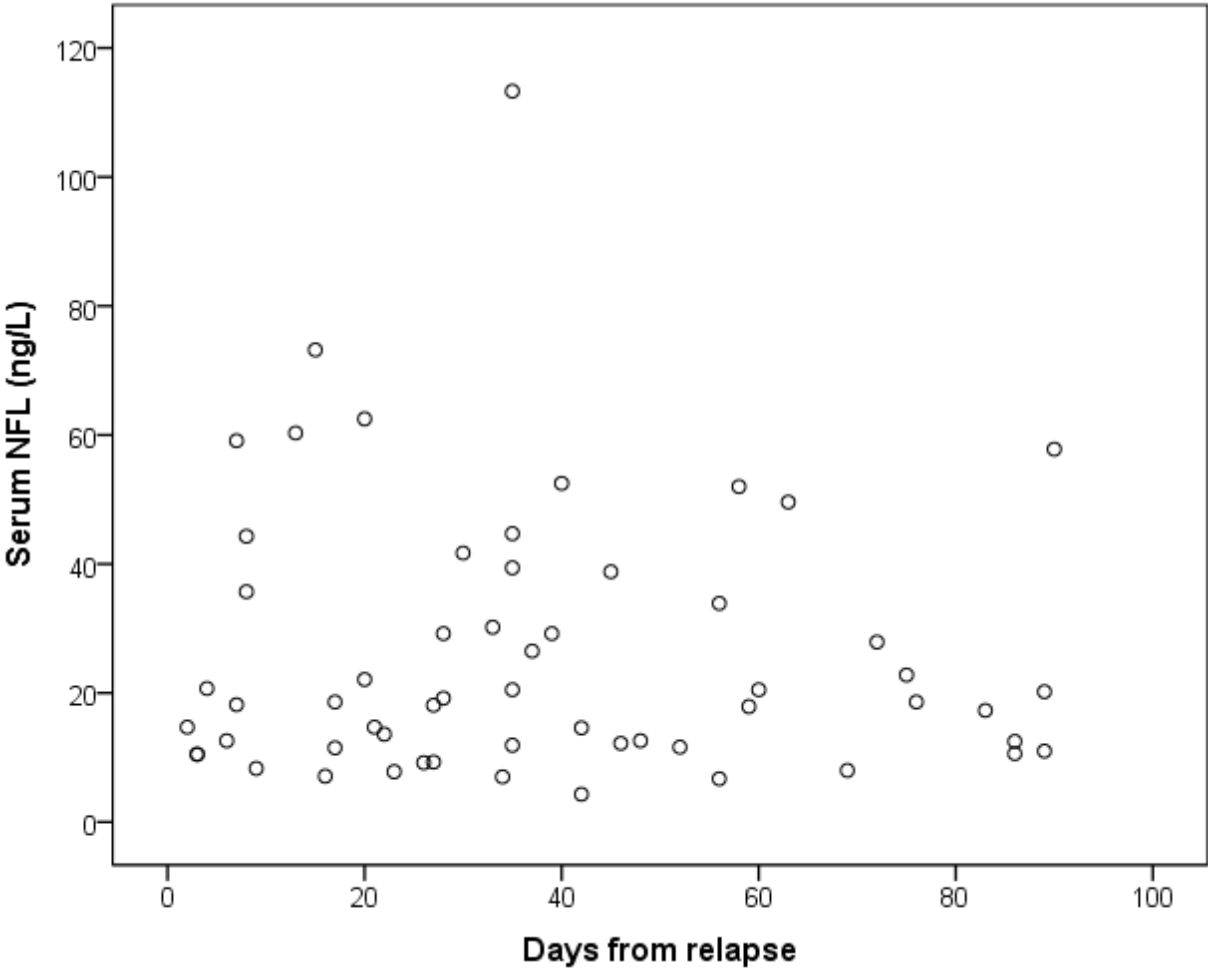

Supplement: Data Supplement [file supp_WNL.0000000000004683_Figure_e-2.pdf]
